# Hounsfield unit value has null effect on thyroid nodules at ^18^F-FDG PET/CT scans

**DOI:** 10.20945/2359-3997000000063

**Published:** 2018-08-01

**Authors:** Filiz Eksi Haydardedeoglu, Gulay Simsek Bagir, Nese Torun, Emrah Kocer, Mehmet Reyhan, Melek Eda Ertorer

**Affiliations:** 1 Baskent University Baskent University Faculty of Medicine Department of Endocrinology and Metabolism Adana Turkey Baskent University Faculty of Medicine Department of Endocrinology and Metabolism, Adana, Turkey; 2 Baskent University Baskent University Faculty of Medicine Department of Nuclear Medicine Adana Turkey Baskent University Faculty of Medicine Department of Nuclear Medicine, Adana, Turkey; 3 Baskent University Baskent University Faculty of Medicine Department of Pathology Adana Turkey Baskent University Faculty of Medicine Department of Pathology, Adana, Turkey

**Keywords:** Thyroid nodules, malignancy, PET-CT, SUV_max_, Hounsfield Unit

## Abstract

**Objectives:**

Detection rate of thyroid nodules is increasing with the use of new imaging modalities, especially in screening for malignancies. Positron emission tomography/computed tomography (PET/ CT)-positive thyroid nodules should be differentiated for malignancy to avoid unnecessary operations and further follow-up. Most trials evaluate the role of SUV_max_, but there is no definitive information about the utility of Hounsfield unit (HU) values for prediction of malignancy. This study aimed to evaluate the HU values beside SUV_max_ for detecting malignancy risk of PET/CT-positive thyroid nodules.

**Subjects and methods:**

Results of 98 cancer patients who had fine needle aspiration biopsy (FNAB) for thyroid nodules detected on PET/CT between January 2011 and December 2015 were assessed. The FNABs and surgical pathological results were recorded.

**Results:**

FNABs revealed benign results in 32 patients (32.7%), malignant in 18 (18.4%), non-diagnostic in 20 (20.4%), and indeterminate in 28 (28.5%). Twenty-four patients underwent thyroidectomy. The mean HU values were not significantly different in benign and malignant nodules (p = 0.73). However, the mean SUV_max_ was significantly higher (*p* < 0.001) in malignant ones. Area under curve (AUC) was 0.824 for SUV_max_; the cut-off value was over 5.55 (*p* < 0.001), with 80% sensitivity, 84.5% specificity.

**Conclusions:**

Our current study demonstrated that HU value does not add any additional valuable information for discriminating between malignant and benign thyroid nodules. We also defined a SUV cut-off value of 5.55 for malignant potential of thyroid nodules detected on PET/CT Arch Endocrinol Metab. 2018;62(4):460-5

## INTRODUCTION

The detection rate of incidental thyroid nodules is increasing steadily due to the use of new radiological imaging modalities, such as ^18^F-fluorodeoxyglucose (FDG) positron emission tomography/computed tomography (PET/CT). Uptake of ^18^F-FDG by the thyroid gland can be detected either diffusely or focally. Diffuse ^18^F-FDG uptake is usually due to benign processes, such as thyroiditis, while focal uptake can be due to either a benign or a malignant nodule. The detection rate of new thyroid nodules by PET/CT has been reported as 1-4% (1).

In the absence of a familial history of thyroid cancer or external beam radiation to the neck, the malignancy rates of thyroid nodules detected on PET/CT ranges between 27.8-74%, whereas it is only 5-13% using ultrasound, CT, or magnetic resonance imaging (MRI) (1–3). Malignant cells tend to have higher glucose metabolism and thus may have positive ^18^F-FDG PET/CT scans. Although they tend to have higher maximum standardized uptake values (SUV_max_) than benign nodules, the definitive cutoff SUV_max_ for the prediction of a malignant thyroid nodule has not yet been defined (4).

The Hounsfield unit (HU), which was first introduced by Sir Godfrey Newbold Hounsfield, is used in CT scans and is a quantity proportional to the degree of X-rays that pass through or are absorbed by tissues (5). HU have since been used to evaluate and quantify tissues and fluids. The radiodensity of water is defined as 0, fat has a negative HU, and blood and other tissues have a positive HU, which are measured and reported in a variety of clinical approaches (6). Thus far, there is no clear information about the utility of HU values for the prediction of thyroid malignancy.

^18^F-FDG PET/CT-positive thyroid nodules tend to have higher rates of malignancy, which should be further investigated by thyroid ultrasonography to enlightment for the features of thyroid nodules. Clinically suspicious features of malignancy on thyroid ultrasonography are hypoechogenicity, taller than wider appearance, irregular margins, hypervascularity, and microcalcifications (7). Many authorities, including the American Thyroid Association (ATA), recommend fine needle aspiration biopsy (FNAB) for exclusion of malignancy in thyroid nodules (8). Although FNAB is a simple, easily performed procedure for the detection of malignancy, in 15-30% of cases the results can be inconclusive. In most cases, repeat FNAB or diagnostic surgery needs to be carried out. More accurate and less invasive diagnostic approaches are required. The aim of this retrospective study is to evaluate the use of SUV_max_ and HU values for detecting the malignancy risk of ^18^F-FDG PET/CT-positive thyroid nodules.

## SUBJECTS AND METHODS

We retrospectively investigated the medical records of 98 patients with various cancers who had FNAB performed following the detection of 18F-FDG PET/ CT-positive thyroid nodules between January 2011 and December 2015. None of the patients in our study group were evaluated for thyroid cancer as a primary site. The study was approved by our Institutional Review Board and Ethics Committee (Project number: KA16/23).

Medical records of all cancer patients with ^18^F-FDG PET/CT-positive thyroid nodules subjected to FNAB were extracted from the hospital database. The ^18^F-FDG PET/CT procedures were performed for the staging and/or follow-up of a cancer. The ultrasonographic features of these nodules were also noted.

The FNAB examinations were reported according to the Bethesda system for thyroid cytopathology (9). According to the Bethesda system, the cytopathology of thyroid nodules is separated into six groups: 1) nondiagnostic or unsatisfactory, 2) benign, 3) atypia of undetermined significance or follicular lesion of undetermined significance, 4) follicular neoplasm or suspicious for a follicular neoplasm, 5) suspicious for malignancy, and 6) malignant. For this particular study, the FNABs were grouped as: 1) nondiagnostic, 2) benign, 3) malignant and suspicious for malignancy, and 4) indeterminate. The indeterminate group included follicular neoplasm/suspected follicular neoplasm, or Hürthle cell neoplasm and atypia of undetermined significance subgroups. If more than one nodule was detected on a PET/CT scan, FNAB was performed on the nodule that had the higher SUVmax.

Together with SUV_max_, HU values of the ^18^F-FDG-positive nodules were also calculated as an additional tool to predict malignancy during non-contrast CT scans taken together with PET imaging. Calculations were performed by the same experienced nuclear medicine physician. The ultrasonographic features and cytological and surgical results were compared with SUV and HU of ^18^F-FDG PET/CT-positive nodules.

### Whole body ^18^F-FDG PET/CT imaging

The patients were imaged using a dedicated PET/CT system (General Electric Medical System, Milwaukee, WI, USA). Patients fasted for at least 6 hours before intravenous administration of 2.5 MBq/kg ^18^F-FDG. Before ^18^F-FDG injection, blood glucose concentration was measured to confirm that patient levels were below 150 mg/dL. During the distribution phase, the patients were kept in the supine position in a quiet room. Combined image acquisition was performed 60 minutes after ^18^F-FDG administration. First, an unenhanced CT scan (3.3 mm slice thickness) from the base of the skull to the inferior border of the pelvis was acquired using a standardized protocol (140 kV and 80 mA). The subsequent PET scan was obtained in three-dimensional mode from the base of the skull to the inferior border of the pelvis (6-7 bed positions, 2.5 minutes per bed position) without repositioning the patient on the table. Patients were breathing shallowly while CT and PET images were acquired. Attenuation was corrected by the CT images obtained after ^18^F-FDG injection.

Thyroid lesions were analyzed semiquantitatively according to SUV values. The SUV was calculated automatically by software, as the ratio of the maximum tissue concentration of FDG (kBq/mL) in the structure delineated by the region of interest (ROI) to the activity injected per gram body weight of the patient (kBq/g). An ^18^F-FDG PET-CT-positive thyroid nodule was defined as a thyroid lesion with a calculated SUV_max_ greater than 2 MBq/kg FDG. The HU value was measured at the region of thyroid nodules marked by a non-contrast CT scan, and calculated as the mean value.

### Statistical analysis

The data are expressed as means ± SD or as medians for data that do not fit a normal distribution. The baseline differences between the two groups were analyzed by Student's *t*-test. Pearson's chi-square test, and Fisher's exact test were used to compare the ratios between groups. The baseline differences between groups were also analyzed by the Kruskal-Wallis test and Mann-Whitney U test. In this study, the cut-off value of SUV_max_ was defined. The sensitivity and specificity of the calculated SUV_max_ and the area under the receiver operating characteristic curve (AUROC) were also measured. A *p* value < 0.05 was considered statistically significant. All statistical analyses were performed using SPSS for Windows software (ver. 23.0; SPSS Inc., Chicago, IL, USA).

## RESULTS

Ninety-eight patients were included in the study. The mean age of the study population (n = 98) was 57.6 ± 13.8 years, and 75 (76.5%) were women. The most common primary malignancy evaluated was breast cancer at 25.5%. Thirty-four patients had solitary nodules (34.7%); the remainder had two or more nodules. Mean maximum diameter of 18F-FDG PET/ CT-positive thyroid nodules was 22.5 ± 11.8 mm. The general characteristics and ultrasonographic features of the patients are shown in detail in Table 1.

**Table 1:** General characteristics of the patients, ultrasonographic features of ^18^F-FDG PET/CT positive nodules regarding FNAB results

	Benign (n = 32)	Malignant (n = 18)	Indeterminated (n = 28)	Non-diagnostic (n = 20)	*p* value
Age (year)	56.6 ± 14.4	53.55 ± 20.93	57.42 ± 11.61	62.2 ± 10.5	0.15
Female (n, %)	27 (36%)	11 (14.6%)	21 (28%)	16 (21.4%)	NS
Male	5 (21.7%)	7 (30.4%)	7 (30.4%)	4 (17.4%)	NS
Type of nodule					
Solid	24 (33.8%)	8 (11.3%)	21 (29.5%)	18 (25.4%)	0.39
Solid-cystic	4 (44.4%)	1 (11.1%)	2 (22.2 %)	2 (22.2%)	0.29
Size (max diam.mm)	22.03 ± 10.5	21.46 ± 13.73	21.93 ± 11.47	22.5 ± 12.71	0.65
Hypoechogenic	18 (32.7%)	14 (25.5%)	13 (23.6%)	10 (18.2%)	0.52
Hyperechogenic	1 (20%)	1 (20%)	2 (40%)	1 (20%)	0.67
Isoechogenic	10 (50%)	1 (5%)	6 (30%)	3 (15%)	0.27
Microcalcification	2 (25%)	1 (12.5%)	0	5 (62.5%)	**0.034**
Taller than wider	0	1 (33.3%)	2 (66.7%)	0	0.32
Vascularity	3 (30%)	3 (30%)	4 (40%)	0	0.23

NS: not significant.

**Figure 1:**
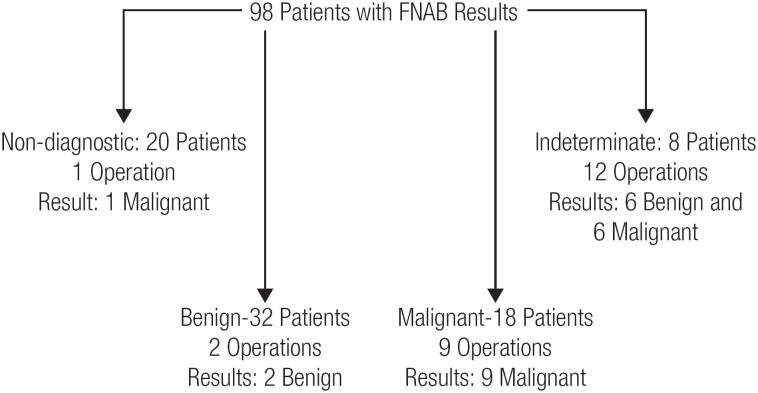
The surgical outcomes of the patients who underwent thyroidectomy.

Cytological examination of FNABs were reported as benign in 32 patients (32.7%), malignant in 18 (18.4%), nondiagnostic in 20 (20.4%), and indeterminate in 28 (28.5%). Thyroidectomy was performed on 24 patients. One of 20 patients in the nondiagnostic FNAB group had surgery, and the nodule was found to be malignant. Two of 32 patients with benign cytology were operated on and were found to have benign nodules. Nine of 18 patients with malignant and suspicious for malignancy FNABs were operated on, and all operation specimens were found to be malignant. The remaining 9 patients with malignant FNABs were not subjected to thyroidectomy because of their accompanying poor general health. Twelve of 28 patients with indeterminate FNABs were subjected to thyroidectomy; 6 of them were diagnosed as malignant, and the remaining were diagnosed as benign. The final pathological examination of 24 cases subjected to thyroidectomy revealed one patient with thyroid metastasis of primary cancer, one with anaplastic thyroid carcinoma, one with follicular carcinoma of thyroid, 13 patients with papillary carcinoma of thyroid, and the remaining eight were diagnosed as benign. These results are summarized in Figure 1.

**Figure 2:**
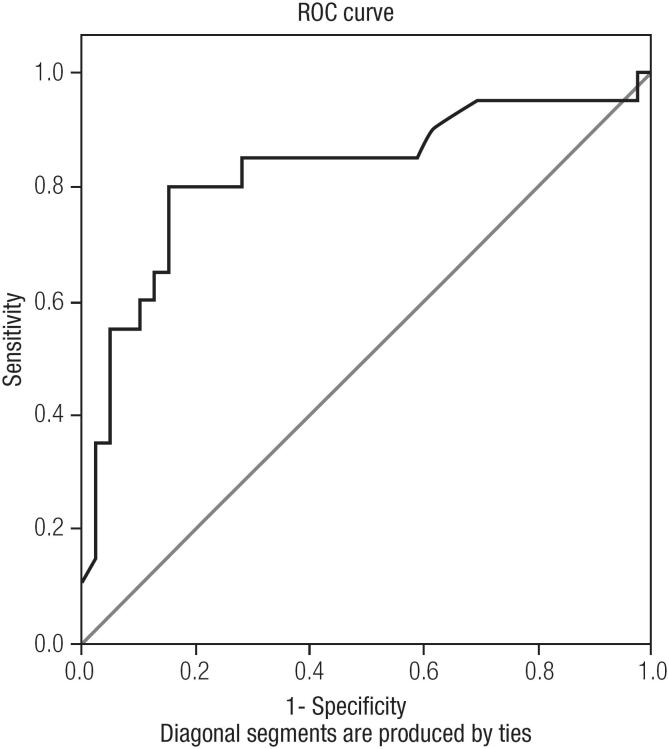
Receiver operating characteristic (ROC) curve of standardized uptake value (SUV max). The sensitivity and specifity in the prediction of malignant thyroid nodules.

When we evaluate FNAB and postoperative pathological results of nodules together, 38 of them were considered as benign (32 patients with benign FNAB results and 6 of 12 patients with indeterminate FNAB results who subjected to thyroidectomy) and 25 of them were malignant (18 cases with malignant FNAB results and 6 cases with indeterminate FNAB results who subjected to thyroidectomy) and one with non-diagnostic FNAB result who subjected to thyroidectomy. The mean SUV_max_ was found to be significantly higher (p < 0.001) in malignant nodules than benign ones: 17.94 (min = 2.1, max = 82.2, median = 11.3, SD = 19.10) versus 4.68 (min = 2, max = 22.4, median = 3.3, SD = 4.02). The under the curve (AUC) was 0.824 for SUV_max_; the cut-off value was over 5.55 (*p* < 0.001), with 80% sensitivity and 84.5% specificity (ROC curve) (Figure 2). However, mean HU values of these malignant and benign thyroid nodules were not significantly different (*p* = 0.73), 50.5 (min = 15, max = 91, median = 49, SD = 16.93) and 43.9 (min = 12, max = 67, median = 51, SD = 17.25), respectively (Table 2). We also grouped the patients according to SUV_max_ cut-off level (5.55). We found that SUV was correlated to HU values in all patients (*p* = 0.03). The nodules that have higher SUV_max_ have higher HU values. However, when only benign and malignant nodules were evaluated, we did not find any correlations between SUV and HU values.

**Table 2:** SUV max and Hounsfield values of ^18^F-FDG PET/CT positive nodules regarding FNAB and postoperative pathological results

	Benign (n = 38)	Malignant (n = 25)	*p* value
SUV max	4.68 ± 4.02	17.94 ± 19.10	
	min = 2	min = 2.1	**< 0.001**
	max = 22.4	max = 82.2	
	median = 3.3	median = 11.3	
Hounsfield	43.9 ± 17.25	50.5 ± 16.93	
Unit	min = 12	min = 15	0.73
	max = 67	max = 91	
	median = 51	median = 49	

SUV max: Maximum Standardized Uptake Value.

## DISCUSSION

In this study, inquiring the malignancy risk of 18F-FDG PET/CT-positive thyroid nodules via using SUV_max_ and HU values, we found that SUV_max_ values of nodules with malignant pathology were higher. However, the HU values exhibited statistically insignificant difference.

The malignancy risk of thyroid nodules ranges between 5-15% (10), and the risk increases if they are detectable on PET/CT scans 27.8-74% (3). Although the prevalence varies widely, according to a recent meta-analysis, 34.8% of thyroid nodules detected by 18F-FDG PET/CT are malignant (11). Even though, only 24.5% of the cases were subjected to thyroidectomy due to their inconvenient general health conditions, keeping in accordance with the analysis above, the malignancy rate was 25.5% in our cohort. The use of the SUV as a semiquantitative parameter to discriminate between benign and malignant tumors has been suggested previously (12,13). This measure reflects the metabolic activity of the lesions and, in general, a higher SUV_max_ value may imply malignancy (14). One of the factors affecting SUV_max_ is the expression of glucose transporters, and thyroid cancer cells usually have increased GLUT-1 expression (12). It has also been suggested that the SUV_max_ is influenced by different grades of inflammation, blood flow, and the size of the malignant lesions (15). Because of the high risk of malignancy, it is important to identify malignant 18F-FDG-positive nodules and determine which patients require surgical intervention. However, there is inconsistent information about the diagnostic role of SUV_max_ values for determining malignancy. Some studies have shown no significant difference between the SUV_max_ values of benign and malignant thyroid nodules (16–18), whereas numerous others have suggested its usefulness for identifying malignant lesions (14,19,20). The importance of SUV_max_ and its definitive cut-off value for predicting malignancy have not yet been established among patients with 18F-FDG PET/CT-positive thyroid nodules. The cut-off value for SUV_max_ was found to be 5.55 in the current study, which is corroborated by a previous report (21). The inconsistent results in current medical literature may be due to differences in glucose metabolism among detected lesions and/or differences in tumor size. Accordingly, Kim and cols. reported no significant difference in SUV_max_ between malignant and benign thyroid nodules less than 1 cm in size (22). When the nodules in our study were categorized up to 1 cm size cut-off, there was no significant difference regarding SUV_max_ between malignant and benign nodules, as well.

To avoid unnecessary surgical operations and further investigations, an additional diagnostic tool besides SUV_max_ is required. Recently, Kim and cols. demonstrated that the HU values of thyroid nodules detected on 18F-FDG PET/CT scans were higher in malignant than benign ones, and the sensitivity, specificity, and accuracy of HU values were all higher than those of SUV_max_ (22). This is the only study proposing the use of HU value as a new parameter to classify the risk of malignancy of 18F-FDG PET/ CT-positive thyroid nodules. For confirmation, we calculated the HU values of thyroid nodules on noncontrast CT in addition to SUV_max_. There was no significant difference between mean HU value of benign and malignant thyroid nodules in our study. The HU measurement seems to have no additional value for the identification of malignant and benign thyroid nodules detected on PET/CT scans. Our finding is consistent with the study by Sayman and cols. (23), although conflicting with Kim's study (22). Further studies are required to verify these results.

Our study has some limitations. The retrospective design is one of them. The other is the relatively low number of cases that were subjected to thyroidectomy in indeterminate and non-diagnostic groups. Only one of the 20 patients with nondiagnostic FNAB, and 12 of the 28 patients with indeterminate FNAB, had thyroidectomy. The final pathological examination showed one malignancy in the former group, whereas 6 cases exhibited thyroid malignancy following thyroidectomy in the indeterminate FNAB group. If all of the nondiagnostic and indeterminate results could have been confirmed by repeat FNAB or surgery, the results would have been surely higher. However, this was not possible due to the critical health condition of the patients (most of whom were metastatic). If the cut-off value for SUV_max_ of 5.55 was applied to nondiagnostic thyroid nodules, 9 of 20 patients would have been above that value. If the same cut-off was applied to the indeterminate patients who did not undergo operation, half of them would have been over that value. These probabilities do not necessarily mean that these nodules were malignant, but suggest that the nodules should have been evaluated further.

In conclusion, we defined an SUV_max_ cut-off value of 5.55 and demonstrated its importance in determining the malignant potential of thyroid nodules. Most of the published research on the issue of thyroid incidentaloma detected at 18F-FDG/PET CT focus on the role of SUV_max_ and its potential role in the differentiation of malignant and benign thyroid lesions. Our study deals with the potential role of additional information obtained from the CT part of this imaging modality. The analysis of HUs of metabolically active thyroid nodules does not have any additional valuable information for discriminating between malignant and benign nodules.

## References

[1] Chun AR, Jo HM, Lee SH, Chun HW, Park JM, Kim KJ, et al. Risk of Malignancy in Thyroid Incidentalomas Identified by Fluorodeoxyglucose-Positron Emission Tomography. Endocrinol Metab (Seoul). 2015;30(1):71-7. 10.3803/EnM.2015.30.1.71 PMC4384680 25325277

[2] Russ G, Leboulleux S, Leenhardt L, Hegedüs L. Thyroid incidentalomas: epidemiology, risk stratification with ultrasound and workup. Eur Thyroid J. 2014;3(3):154-63. 10.1159/000365289 PMC4224250 25538897

[3] Kao YH, Lim SS, Ong SC, Padhy AK. Thyroid incidentalomas on fluorine-18-fluorodeoxyglucose positron emission tomography-computed tomography: incidence, malignancy risk, and comparison of standardized uptake values. Can Assoc Radiol J. 2012;63(4):289-93. 10.1016/j.carj.2011.04.003 22136969

[4] Brindle R, Mullan D, Yap BK, Gandhi A. Thyroid incidentalomas discovered on positron emission tomography CT scanning - Malignancy rate and significance of standardised uptake values. Eur J Surg Oncol. 2014;40(11):1528-32. 10.1016/j.ejso.2014.05.005 24915858

[5] Lamba R, McGahan JP, Corwin MT, Li CS, Tran T, Seibert JA, et al. CT Hounsfield numbers of soft tissues on unenhanced abdominal CT scans: variability between two different manufacturers' MDCT scanners. AJR Am J Roentgenol. 2014;203(5):1013-20. 10.2214/AJR.12.10037 PMC5015439 25341139

[6] Kalender WA. Computed tomography: fundamentals, system technology, image quality, applications. 3. Erlangen, Germany: Publicis Publishing; 2011.

[7] Yoon JH, Cho A, Lee HS, Kim EK, Moon HJ, Kwak JY. Thyroid incidentalomas detected on 18F-fluorodeoxyglucose-positron emission tomography/computed tomography: Thyroid Imaging Reporting and Data System (TIRADS) in the diagnosis and management of patients. Surgery. 2015;158(5):1314-22. 10.1016/j.surg.2015.03.017 25958065

[8] Haugen BR, Alexander EK, Bible KC, Doherty GM, Mandel SJ, Nikiforov YE, et al. 2015 American Thyroid Association Management Guidelines for Adult Patients with Thyroid Nodules and Differentiated Thyroid Cancer: The American Thyroid Association Guidelines Task Force on Thyroid Nodules and Differentiated Thyroid Cancer. Thyroid. 2016;26(1):1-133. 10.1089/thy.2015.0020 PMC4739132 26462967

[9] Cibas ES, Ali SZ. The Bethesda System for Reporting Thyroid Cytopathology. Thyroid. 2009;19(11):1159-65. 10.1089/thy.2009.0274 19888858

[10] Tunbridge WM, Evered DC, Hall R, Appleton D, Brewis M, Clark F, et al. The spectrum of thyroid disease in a community: the Whickham survey. Clin Endocrinol (Oxf). 1977;7(6):481-93. 10.1111/j.1365-2265.1977.tb01340.x 598014

[11] Soelberg KK, Bonnema SJ, Brix TH, Hegedüs L. Risk of malignancy in thyroid incidentalomas detected by 18F-fluorodeoxyglucose positron emission tomography: a systematic review. Thyroid. 2012;22(9):918-25. 10.1089/thy.2012.0005 22827552

[12] Chen YK, Ding HJ, Chen KT, Chen YL, Liao AC, Shen YY, et al. Prevalence and risk of cancer of focal thyroid incidentaloma identified by 18F-fluorodeoxyglucose positron emission tomography for cancer screening in healthy subjects. Anticancer Res. 2005;25(2B):1421-6. 15865100

[13] Yaylali O, Kirac FS, Yuksel D, Marangoz E. Evaluation of focal thyroid lesions incidentally detected in fluorine-18-fluorodeoxyglucose positron emission tomography/computed tomography images. Indian J Cancer. 2014;51(3):236-40. 10.4103/0019-509X.146737 25494112

[14] Stangierski A, Wolinski K, Czepczynski R, Czarnywojtek A, Lodyga M, Wyszomirska A, et al. The usefulness of standardized uptake value in differentiation between benign and malignant thyroid lesions detected incidentally in 18F-FDG PET/CT examination. PLosOne. 2014; 9(10):e109612. 10.1371/journal.pone.0109612 PMC4190406 25296297

[15] Ohba K, Nishizawa S, Matsushita A, Inubushi M, Nagayama K, Iwaki H, et al. High incidence of thyroid cancer in focal thyroid incidentaloma detected by 18F-fluorodeoxyglucose [corrected] positron emission tomography in relatively young healthy subjects: results of 3-year follow-up. Endocr J. 2010;57(5):395-401. 10.1507/endocrj.k10e-008 20160400

[16] Eloy JA, Brett EM, Fatterpekar GM, Kostakoglu L, Som PM, Desai SC, et al. The significance and management of incidental [18F] fluorodeoxyglucose-positron-emission tomography uptake in the thyroid gland in patients with cancer. AJNR Am J Neuroradiol. 2009;30(7):1431-4. 10.3174/ajnr.A1559 PMC7051573 19342543

[17] Buyukdereli G, Aktar Y, Kara E, Uguz A, Sonmez H. Role of 18F-fluorodeoxyglucose Positron Emission Tomography/Computed Tomography in the Evaluation of Cytologically Indeterminate Thyroid Nodules. Iran J Radiol. 2016;13(1):e21186. 10.5812/iranjradiol.21186 PMC4837297 27110335

[18] Are C, Hsu JF, Schoder JP, Shah JP, Larson SM, Shaha AR. FDG-PET detected thyroid incidentalomas: need for further investigation? Ann Surg Oncol. 2007;14(1):239-47. 10.1245/s10434-006-9181-y 17024553

[19] Wang N, Zhai H, Lu Y. Is fluorine-18 fluorodeoxyglucose positron emission tomography useful for the thyroid nodules with indeterminate fine needle aspiration biopsy? A meta-analysis of the literature. J Otolaryngol Head Neck Surg. 2013;42:38. 10.1186/1916-0216-42-38 PMC3765697 24228840

[20] Kresnik E, Gallowitsch HJ, Mikosch P, Stettner H, Igerc I, Gomez I, et al. Fluorine-18-fluorodeoxyglucose positron emission tomography in the preoperative assessment of thyroid nodules in an endemic goiter area. Surgery. 2003;133(3):294-9. 10.1067/msy.2003.71 12660642

[21] Bertagna F, Treglia G, Piccardo A, Giovannini E, Bosio G, Biasiotto G, et al. F18-FDG-PET/CT thyroid incidentalomas: a wide retrospective analysis in three Italian centres on the significance of focal uptake and SUV value. Endocrine. 2013;43(3):678-85. 10.1007/s12020-012-9837-2 23179777

[22] Kim D, Hwang SH, Cha J, Jo K, Lee N, Yun M. Risk Stratification of Thyroid Incidentalomas Found on PET/CT: The value of Iodine Content on Noncontrast Computed Tomography. Thyroid. 2015;25(11):1249-54. 10.1089/thy.2015.0200 26335604

[23] Sayman H, Uslu L, Topuz OV, Sager S, Halac M, Sonmezoglu K. Is adding hounsfield unit measurement on standardized uptake value helpful in differentiating thyroid nodules? J Nucl Med. 2013;54(Suppl 2):1900.

